# Computational Prediction of Thrombosis in Food and Drug Administration’s Benchmark Nozzle

**DOI:** 10.3389/fphys.2022.867613

**Published:** 2022-04-25

**Authors:** Yonghui Qiao, Kun Luo, Jianren Fan

**Affiliations:** ^1^ State Key Laboratory of Clean Energy Utilization, Zhejiang University, Hangzhou, China; ^2^ Shanghai Institute for Advanced Study of Zhejiang University, Shanghai, China

**Keywords:** thrombosis, backward-facing step, FDA benchmark nozzle, platelet consumption, computational fluid dynamics

## Abstract

Thrombosis seriously threatens human cardiovascular health and the safe operation of medical devices. The Food and Drug Administration’s (FDA) benchmark nozzle model was designed to include the typical structure of medical devices. However, the thrombosis in the FDA nozzle has yet not been investigated. The objective of this study is to predict the thrombus formation process in the idealized medical device by coupling computational fluid dynamics and a macroscopic hemodynamic-based thrombus model. We developed the hemodynamic-based thrombus model by considering the effect of platelet consumption. The thrombus model was quantitatively validated by referring to the latest thrombosis experiment, which was performed in a backward-facing step with human blood flow. The same setup was applied in the FDA nozzle to simulate the thrombus formation process. The thrombus shaped like a ring was firstly observed in the FDA benchmark nozzle. Subsequently, the accuracy of the shear-stress transport turbulence model was confirmed in different turbulent flow conditions. Five scenarios with different Reynolds numbers were carried out. We found that turbulence could change the shape of centrosymmetric thrombus to axisymmetric and high Reynolds number blood flow would delay or even prevent thrombosis. Overall, the present study reports the thrombosis process in the FDA benchmark nozzle using the numerical simulation method, and the primary findings may shed light on the effect of turbulence on thrombosis.

## Introduction

Cardiovascular disease has become the number one threat to human life in the world (Yusuf et al., 2015). Thrombus is associated with some common cardiovascular diseases, such as myocardial infarction and stroke. Typically, efforts are taken to prevent thrombosis in cardiovascular and blood-coated medical devices. It should be noted that the thrombus is sometimes expected to form in specific situations, and the degree of thrombosis in the false lumen is a key indicator for evaluating the surgical outcomes of aortic dissection (Nienaber et al., 2013). There is an urgent need to accurately predict macroscopic thrombosis for assessing disease risk and improving the performance of medical devices.

Several attempts have been made to reveal the complex formation mechanism of macroscopic thrombus. Taylor et al. (2014) conducted *in vitro* thrombosis experiments using bovine blood in a backward-facing step (BFS) model and macroscopic data on thrombus were obtained with magnetic resonance imaging (MRI) technology. Subsequently, they developed a computational thrombosis model and correctly reproduced the location of device-induced thrombosis including the result of the above experiment (Taylor et al., 2016b). Almost simultaneously, Menichini and Xu (2016) also proposed a novel hemodynamics-based thrombosis model, which has been applied to predict false lumen thrombosis in type B aortic dissection and the computational results were in qualitative agreement with *in vivo* observations (Menichini et al., 2016; Menichini et al., 2018). In our previous study, thrombosis risk in the left atrium under atrial fibrillation was investigated by using Menichini’s hemodynamics-based thrombosis model (Wang et al., 2020). Additionally, we proposed a reduced-order fluid-chemical model to rapidly predict the coagulation cascade, which provides a potential clinical application (Wang et al., 2021). Recently, Yang et al. (2020) provided experimental data for device-induced thrombosis using bovine and human blood in the same backward-facing step model as Taylor et al. (2014). Besides, Yang et al. (2021) analyzed the thrombosis formation for non-Newtonian blood by developing the device-induced thrombosis model proposed by Taylor et al. (2016b). These studies have laid a good foundation for macroscopic thrombosis research.

For validating novel biomedical computational fluid dynamics (CFD) methodologies, the U.S. Food and Drug Administration (FDA) developed a benchmark nozzle model with a conical shape in diameter at one end of the throat and a sudden expansion at the other end. Particle image velocimetry and laser Doppler velocimetry have been used to measure the transitional and turbulent blood flow in the FDA nozzle (Hariharan et al., 2011; Taylor et al., 2016a). Besides, a considerable amount of computational fluid dynamics literature has been published to validate the numerical models, such as laminar, Reynolds-averaged Navier-Stokes model, large eddy simulation (LES), direct numerical simulation (DNS), and lattice Boltzmann method (Stewart et al., 2012; Bhushan et al., 2013; Delorme et al., 2013; Janiga, 2014; Jain, 2020; Manchester and Xu, 2020; Sánchez Abad et al., 2020). As for the common biological responses including hemolysis and thrombosis, most of the attention has been paid to hemolysis (Trias et al., 2014; Tobin and Manning, 2020). However, thrombosis in the FDA benchmark nozzle has not been explored and little is currently known about the thrombus formation process.

The specific objective of this study is to investigate the thrombosis mechanism in the FDA benchmark nozzle. We first validated the predictive capacity of the hemodynamics-based thrombus model using the latest BFS experimental data. Subsequently, the thrombosis in the FDA nozzle was predicted under the same conditions as the BFS experiment. Finally, the effect of turbulence on thrombosis was explored. This is the first study to report the thrombus formation process in the FDA benchmark nozzle and the findings may make an important contribution to the field of device-induced thrombosis.

## Methodologies

### Thrombosis Model

**FIGURE 1:**
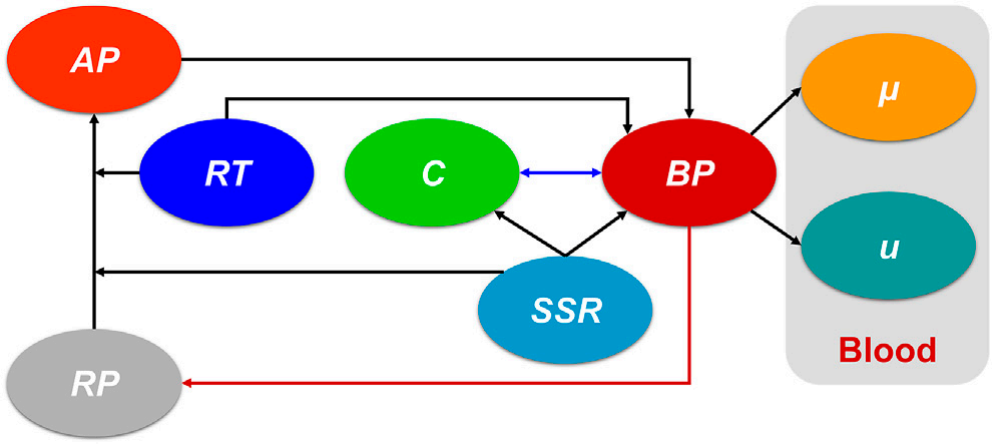
The connections of the main variables in the present hemodynamics-based thrombosis model. The dark blue double arrow line indicates the interaction between the *C* and *BP*. The dark red arrow line denotes the consumption of platelets during thrombosis. *AP*: activated platelet, *RP*: resting platelet, *RT*: residence time, *C*: coagulant, *SSR*: shear strain rate, *BP*: bound platelets, μ and u is the viscosity and velocity of the blood, respectively.

The present study is based on a previous hemodynamics-based thrombosis model (Menichini and Xu, 2016), which was chosen because of its reliability and efficiency. Residence time (*RT*) of blood flow, resting (healthy) and activated platelets (*RP* and *AP*), and coagulant (*C*) were coupled with Navier-Stokes equations to produce the physiological environment for thrombus formation, which could be depicted by the concentration of bound platelets (*BP*). It should be emphasized that the shear strain rate (*SSR*) is a crucial parameter in this thrombosis model. Figure 1 shows the connections of the main variables. The dark blue double arrow line indicates the interaction between the *C* and *BP*. The dark red arrow line denotes the consumption of platelets during thrombosis. The critical governing equations and our contribution are given below:

Residence time (*RT*) of blood flow could capture the regions of stagnancy and flow recirculation. High *RT* means a long stay of platelets and the corresponding convection-diffusion-reaction transport equation is as follows (Ghirelli and Leckner, 2004):

$$\frac{\partial RT}{\partial t}+u\cdot \text{∇}RT=D_{RT}{\text{∇}}^{2}RT+1$$ (1)

where u is the blood flow velocity, and $D_{RT}$ denotes the diffusion coefficient (Harrison et al., 2007).

The distribution of platelets could be acquired by this transport equation (Sorensen et al., 1999):

$$\frac{\partial c}{\partial t}+u\cdot \text{∇}c=D_{p}{\text{∇}}^{2}c+S$$ (2)

where c can represent *RP* and *AP*, and it indicates the corresponding concentration. The diffusion coefficient $D_{p}$ denotes the shear-enhancing effect of red blood cells (Wootton et al., 2001):

$$D_{p}=D_{p\text{t}}+\alpha \;\dot{\gamma }$$ (3)

where $D_{p\text{t}}$ is the thermal diffusivity and α  is a constant, $\dot{\gamma }$ is *SSR*:

$$SSR={\left[2\frac{\partial U_{i}}{\partial x_{j}}S_{ij}\right]}^{\frac{1}{2}}$$ (4)

where $U_{i}$ represents velocity components, $S_{ij}$ is the strain rate tensor:

$$S_{ij}=\frac{1}{2}\left(\frac{\partial U_{i}}{\partial x_{j}}+\frac{\partial U_{j}}{\partial x_{i}}\right)$$ (5)

The source term S describes the transition from *RP* to *AP* under the action of blood flow (Anand et al., 2003):

$$S=k_{1}\;\cdot AP\;\cdot RP+k_{2}\cdot \;RP\;\cdot RTt$$ (6)

where RTt indicates the relative *RT* and the corresponding definition is the ratio of *RT* to simulated time in this study.

RTt=RT/t (7)

The coagulant (*C*) is applied to simplify the complex coagulation cascade and the corresponding transport equation is diffusion-dominated:

$$\frac{\partial C}{\partial t}=D_{ceff}{\text{∇}}^{2}C+k_{C}\phi_{BP}\phi_{\dot{\gamma }}$$ (8)

$$D_{ceff}=\phi_{\dot{\gamma }}\;D_{c}$$ (9)

$$\phi_{BP}=\frac{BP^{2}}{BP^{2}+BP_{t}^{2}}$$ (10)

$$\phi_{\dot{\gamma }}=\frac{{\bar{\dot{\gamma }}}_{\text{t}}^{2}}{{\bar{\dot{\gamma }}}^{2}+{\bar{\dot{\gamma }}}_{\text{t}}^{2}}$$ (11)

where $D_{ceff}$ is the diffusion coefficient and $k_{C}$ is the reaction constant. To artificially accelerate the thrombosis process, the diffusion coefficient is amplified 150 times following the practice of the previous study (Menichini et al., 2016). $\phi_{BP}$ and $\phi_{\dot{\gamma }}$ are introduced to control the start and stop of the reaction. A constant concentration of *C* is specified on the low *SSR* wall to activate the thrombosis model:

$$C_{0}=\{\begin{array}{ll} 1\times 10^{-4}\;mol\;m^{-3} & if\;\;\;\;\bar{\dot{\gamma }}\leq 1\;\;{\text{s}}^{-1}\\ 0 & \text{otherwise} \end{array}$$ (12)

where $BP_{t}$ and ${\bar{\dot{\gamma }}}_{t}$ are the threshold value of *BP* and time-averaged *SSR*, respectively. *BP* is formed in regions with a high value of *RT*, *AP*, and *C*. It should be noted that the governing equation of *BP* only has one source term:

$$\frac{\partial BP}{\partial t}=k_{BP}\phi_{C}\phi_{RRT}\phi_{\dot{\gamma }}\left[AP\right]$$ (13)

$$\phi_{C}=\frac{C^{2}}{C^{2}+C_{t}^{2}}\;\;\;\;\;\;\phi_{RRT}=\frac{RTt^{2}}{RTt^{2}+RTt_{t}^{2}}$$ (14)

where $k_{BP}$ is the reaction constant, $\phi_{C}$ and $\phi_{RRT}$ represent the effect of *C* and RTt , respectively. [*AP*] is the normalized form of *AP* to its initial value (*AP*_*0*_). $C_{t}$ and $RTt_{t}$ are the threshold value of *BP* and *RTt*, respectively.

In the thrombosis region, the viscosity of blood is considered to increase significantly to simulate flow resistance (Goodman et al., 2005). Besides, a negative source term ( $S_{M}$ ) is added to the momentum equations to account for the influence of the thrombus on the blood flow velocity:

$$\mu =\mu_{0}\left(1+100\phi_{BP}\right)$$ (15)

$$S_{M}=K_{M}\phi_{BP}u$$ (16)

where $\mu_{0}$ is the reference value of viscosity and $K_{M}$ is a sufficiently high coefficient to stop the blood flow. Table 1 shows the parameters of the thrombosis model.

**TABLE 1:** Parameters of the thrombosis computational model.

Parameter	Description	Value	References
$D_{RT}$	*RT* diffusion coefficient	1.14 × 10^–11^ m^2^ s^−1^	Harrison et al. (2007)
$D_{p\text{t}}$	$D_{p}$ thermal diffusivity	1.6 × 10^–13^ m^2^ s^−1^	Wootton et al. (2001)
α	constant	7 × 10^–13^ m^2^	Wootton et al. (2001)
$k_{1}\;$	kinetic constant	3 × 10^5^ m^3^ mol^−1^ s^−1^	Anand et al. (2003)
$k_{2}$	kinetic constant	0.5 s^−1^	Anand et al. (2003)
$D_{c}$	*C* diffusion constant	1 × 10^–8^ m^2^ s^−1^	Menichini and Xu (2016)
$k_{C}$	*C* reaction constant	2 × 10^–4^ mol m^−3^ s^−1^	Menichini and Xu (2016)
$BP_{t}$	*BP* threshold	2 × 10^–5^ mol	Menichini and Xu (2016)
${\bar{\dot{\gamma }}}_{t}$	$\bar{\dot{\gamma }}$ threshold	10 s^−1^	Menichini and Xu (2016)
$k_{BP}$	*BP* reaction constant	1 × 10^–5^ mol m^−3^ s^−1^	Menichini and Xu (2016)
*AP*_*0*_	*AP* initial value	5% *P*_*in*_	Menichini and Xu (2016)
$C_{t}$	*C* threshold	1 × 10^–5^ mol	Menichini and Xu (2016)
$RTt_{t}$	RTt threshold	0.9	Menichini and Xu (2016)
$K_{M}$	coefficient	10^7^ kg m^−3^ s	Menichini and Xu (2016)

### Thrombosis Experiment in Backward-Facing Step

**FIGURE 2:**
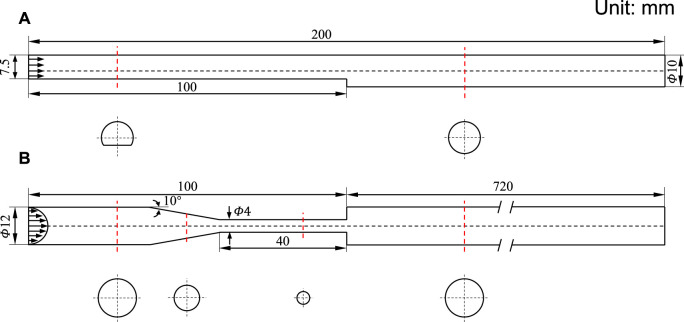
Schematic diagram of 3D geometric models. **(A)** Backward-facing step model. **(B)** Benchmark nozzle of Food and Drug Administration. The inlet arrows indicate the velocity profile. The red dotted line indicates the cross-section position, and the cross-section shape is below.

The state of art thrombosis experiment by Yang et al. (2020) was chosen to validate the accuracy of the thrombus formation model. An experimental flow loop involving the BFS geometry was constructed, and the upstream and downstream lengths are sufficient for the full development of blood flow. The geometric dimensions of the BSF model are shown in Figure 2. A total of 450 ml of human blood with a constant inflow rate of 0.76 L/min was circulated through the loop by a peristaltic pump for 30 min. Real-time 3D MRI was used to capture the shape of the thrombus with Magnevist as the contrast agent.

Results show that the thrombus forms at the flow recirculation region near the step, the length of the thrombus gradually increased to 13.3 ± 0.6 mm during the first 15 min and stabilized after 20 min. The reason for stabilized thrombosis was that the platelets and coagulation factors of the finite recirculating blood in the closed loop were all consumed. Therefore, the number of inlet platelets is real-time controlled in our present study and it decreases as the volume of the thrombus grows. The consumed platelets can be calculated by the following formula:

$$N_{\text{platelet}}^{\text{CFD}}=V_{\text{thrombus}}^{\text{CFD}}\left(N_{\text{platelet}}^{\text{EXP}}/V_{\text{thrombus}}^{\text{EXP}}\right)$$ (17)

where EXP represents the data from the thrombosis experiment and CFD refers to our numerical simulation.

### FDA Benchmark Nozzle

The FDA benchmark nozzle contains four sections: a straight tube inlet, a tapered section, a straight throat section, and a straight tube that suddenly expanded (Figure 2). Based on the inlet velocity profile (defined later), a relatively small length was assigned to the straight tube inlet. We have chosen the outlet length to be 60 times the inlet diameter to avoid backflow. The region of interest is located near the jet inlet, where the thrombus would form according to the result of the thrombosis experiment.

### Computational Details

Based on the thrombosis experiment, the blood was considered a Newtonian fluid with a constant viscosity of 0.0044 Pa s and a density of 1038 kg/m^3^. The number of platelets was 1.3824 × 10^–13^ mol which can be obtained by multiplying the blood volume by the platelets number per unit volume. We assumed that the volume of the thrombus was directly proportional to the number of platelets, so the relationship mentioned above was applied to control the concentration of platelets entering the blood flow field (Eq 17).

For the BFS model, the inlet flow rate was uniform because of the non-circular inlet shape, and a zero averaged pressure was used as the outlet boundary condition. The entrance of the FDA nozzle was circular, so a parabolic velocity profile was imposed at the inlet to reduce the length of the inlet straight pipe (Qiao et al., 2022a; Qiao et al., 2022b):

$$u_{\left(r\right)}=2\frac{Q_{inlet}}{A_{inlet}}\left(1-\frac{r^{2}}{r_{inlet}^{2}}\right)$$ (18)

where r is the radial location of the inlet. The outlet boundary condition of the nozzle remains unchanged. It should be noted that a no-slip boundary condition was applied to the wall of the BFS and FDA nozzle. Based on the experimental inlet velocity of blood flow, the corresponding inlet Reynolds numbers are 472 and 317, respectively, so the blood flow was both assumed to be laminar in the BFS and FDA nozzle. Our simulations were unsteady considering the effect of thrombus on the flow field.

To evaluate the effect of turbulence on thrombosis in the FDA nozzle, we designed four cases by modifying the Reynolds numbers of the throat inlet (Re = 100, 500, 3,500, and 5,000). For turbulent flow, the shear-stress transport turbulence model (SST) was used to predict the blood flow field, which was validated by experimental data (*Computational Details*) (NCI Hub contributors, 2022). An automatic near-wall treatment method was adopted as the wall function (Ansys, 2006).

Both the BFS and FDA nozzle were meshed by using ANSYS-ICEM 16.1 (ANSYS Inc., Canonsburg, PA, United States). The two blood flow domains had more than two and six million hexahedron elements, respectively. We placed ten grid nodes inside the near-wall boundary layer and the first layer near the wall was set to 20 μm to satisfy the requirements of the SST model (y^+^ < 1). All the simulations were performed on ANSYS-CFX (ANSYS Inc., Canonsburg, PA, United States). Sensitivity analyses were carried out and the differences in velocity and *SSR* between the chosen meshes and finer meshes were less than 1%.

## Results

### Thrombosis Model Validation

**FIGURE 3:**
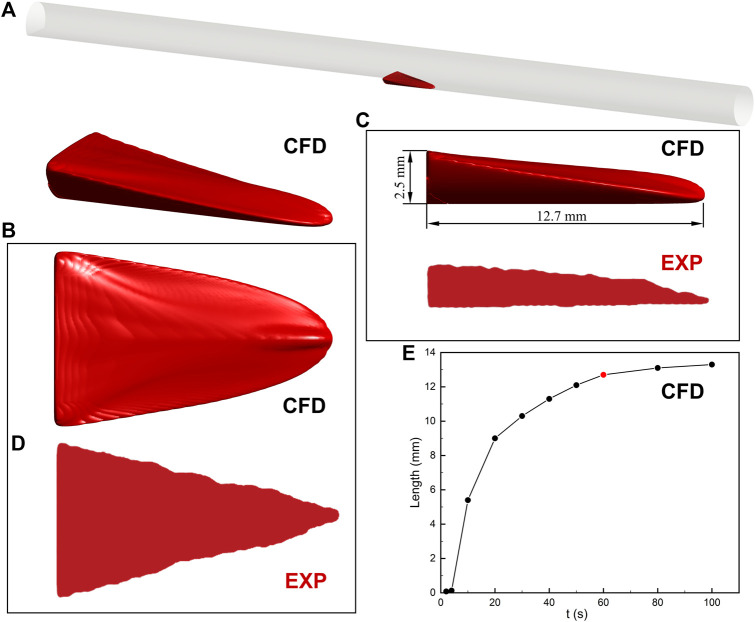
Thrombus formation in the backward-facing step model. **(A)** Schematic diagram of the location of the thrombus. **(B)** 3D thrombus shape. CFD refers to the result of computational prediction. **(C)** Side view with height and length of thrombus. The prediction of the thrombus model is in acceptable agreement with the experimental result (EXP). **(D)** Top view. The difference in the thrombus shape may result from the contrast agent and MRI resolution. **(E)** The relationship between the axial length of thrombus and simulation time. The increase in thrombus length is negligible after 60 s. The insets of the thrombus come from experimental observation (Yang et al., 2020).

Thrombosis in the BFS experiment was predicted by the hemodynamics-based thrombus model (Figure 3). The location of the thrombus was accurately captured and the mathematic model depicted the 3D thrombus shape with comparable height and axial length. There is a slight difference in the top view shapes of the thrombus. The experiment reported a triangular ramp thrombi shape, while we observed a quasi-triangular slope shape. Overall, the prediction of the thrombus model is in acceptable agreement with the latest experimental result.

The relationship between the axial length of the thrombus and simulation time is also investigated in Figure 3. The thrombus almost stopped developing after 60 s due to the platelets no longer entering the blood flow domain in the present study. The increase of thrombus length between the 60 and 80 s is less than 4%. In addition, the axial length of the thrombus in 60 s (12.7 mm) is within the range of experimental measurement (13.3 ± 0.6 mm). Therefore, the prediction result of the thrombus model in 60 s is present in this study.

### Thrombus Formation in FDA Nozzle

**FIGURE 4:**
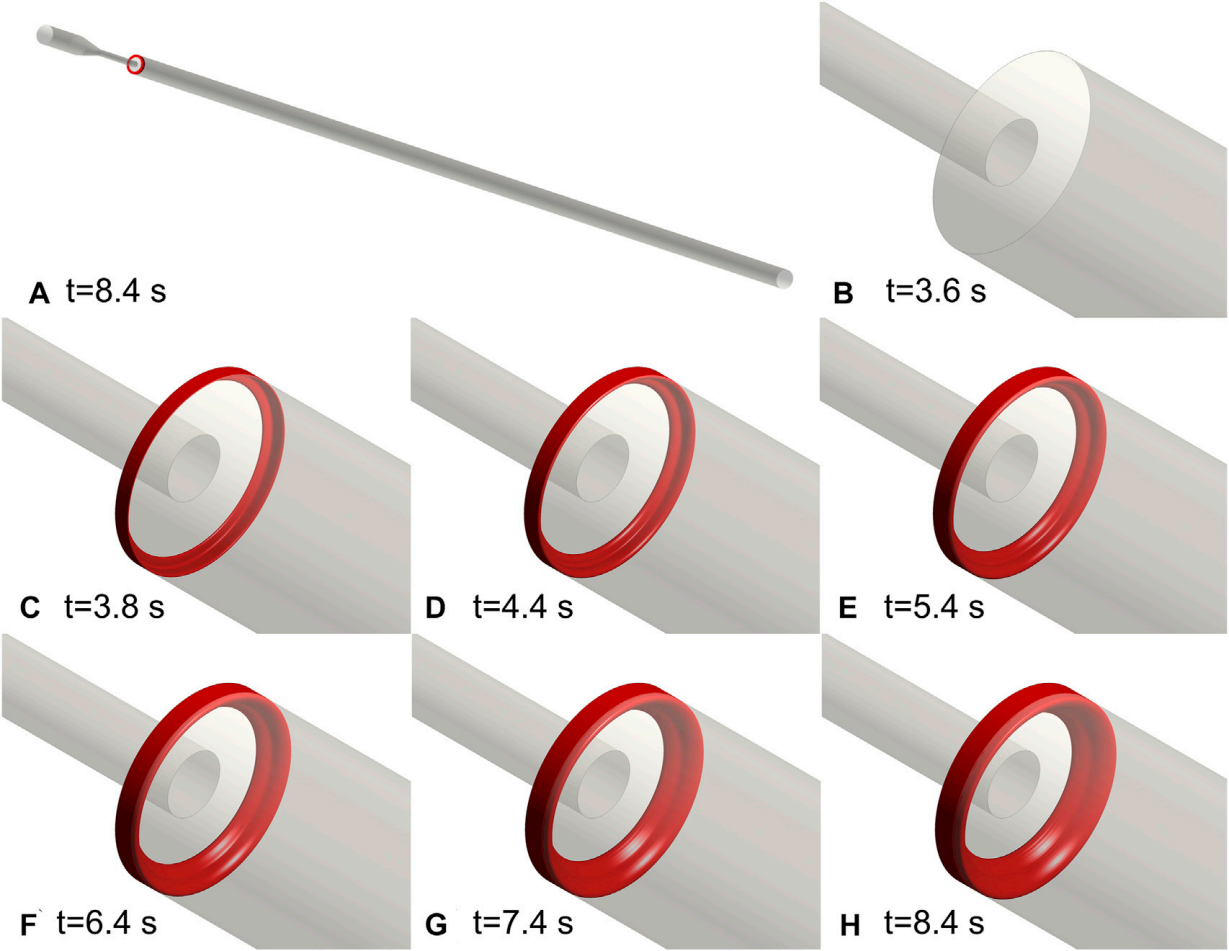
Thrombus formation in the FDA benchmark nozzle. **(A)** Schematic diagram of the location of the thrombus. **(B**–**H)** Annular thrombosis process. All the activated platelets were consumed at 8.4 s and the thrombus reached its maximum volume. The final thrombus is shaped like a ring.

The same setup of the BSF experiment was applied to the FDA nozzle. Figure 4 depicts the process of thrombus formation in the FDA benchmark nozzle. The thrombus locates at the annular corner of the expansion section. The activated platelets were consumed at 8.4 s and the thrombus reached its maximum volume (67.5 mm^3^). The final thrombus is shaped like a ring.

### Validation of Blood Flow Model in FDA Nozzle

**FIGURE 5:**
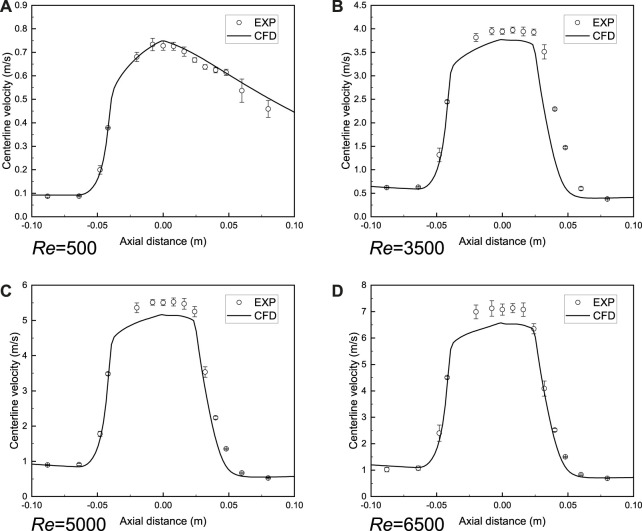
Validation of blood flow model in FDA benchmark nozzle. **(A)** Laminar model. **(B**–**D)** Shear-stress transport turbulence model (SST). The axial velocity along the centerline is close to the experimental measurement result (NCI Hub contributors, 2022) and it is reasonable to adopt the SST model to predict the turbulent blood flow in the FDA nozzle. CFD is the computational result and EXP indicates the experimental result.

The comparison of axial velocity along the centerline between simulation and experiment (NCI Hub contributors, 2022) is shown in Figure 5. The laminar model could accurately reproduce the blood flow when the Reynolds number is relatively low. For turbulent flow, the centerline velocity is close to the experimental measurement result and it is reasonable to adopt the SST model to predict the turbulent blood flow in the FDA nozzle. It should be mentioned that the SST model failed to match with the experimental value when the Reynolds number is 2000. Therefore, the complicated transition process between laminar flow and turbulent flow is not considered in this study.

### Thrombosis and Turbulence

**FIGURE 6:**
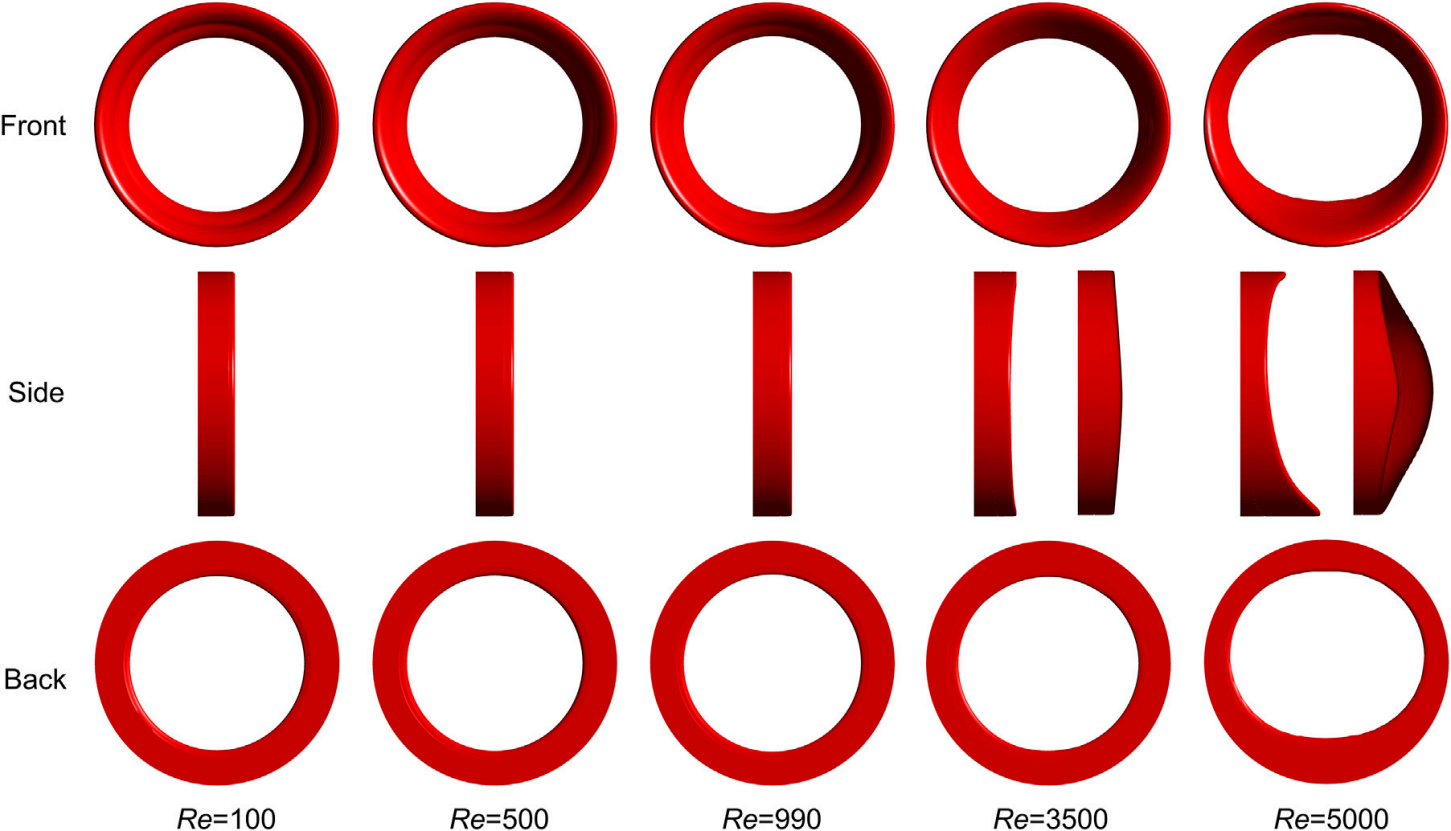
Final thrombus shape in FDA benchmark nozzle. Thrombus volume is the same at different Reynolds numbers. There is no significant difference between the thrombus shapes predicted by the laminar model and the annular thrombus is centrosymmetric. However, the thrombus becomes only axisymmetric when turbulence is present. Three rows indicate three different views. Two side views are used to show the thrombus shapes for Re = 3,500 and 5,000.

Figure 6 illustrates the final thrombus shape in the FDA benchmark nozzle. Thrombus volume is the same at different Reynolds numbers. There is no significant difference between the thrombus shapes predicted by the laminar model (Re = 100, 500, and 990). Considering that the regular annulus has an infinite number of axes of symmetry, we use centrosymmetric to describe the thrombus shape. However, the thrombus becomes irregular and the number of axes of symmetry reduces to two and one, respectively, when the turbulence is present (Re = 3,500 and 5,000). Therefore, we use axisymmetric to describe the thrombus shape. The most interesting finding is that thrombus no longer appears in FDA nozzle when Reynolds number increases to 6,500.

**FIGURE 7:**
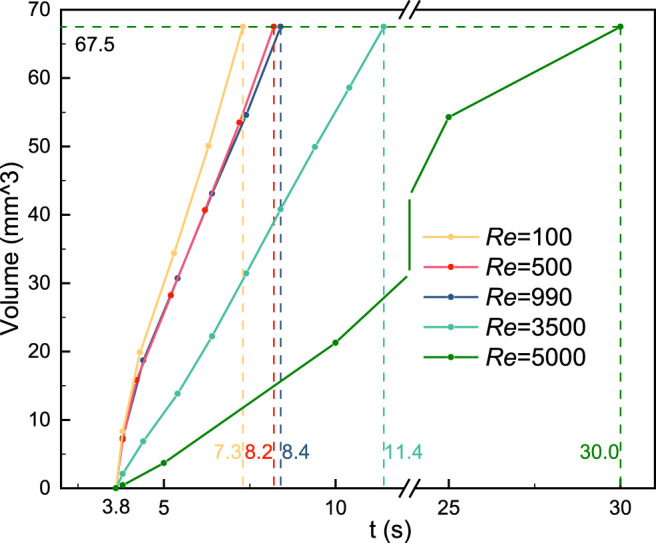
Relationship between the thrombus volume and simulation time. Blood flow with a low Reynolds number would accelerate the thrombus formation. On the contrary, the thrombosis process would be slowed by high Reynolds number and turbulence. The vertical dashed line indicates the respective computational time and the horizontal line is the final thrombus volume.

The relationship between the thrombus volume and simulation time is revealed in Figure 7. The vertical dashed line indicates the respective computational time and the horizontal line is the final thrombus volume. It should be noted that the thrombus begins to form at the same time (3.8 s). Blood flow with a low Reynolds number would accelerate the thrombus formation (Re = 100, 500, and 990). On the contrary, the thrombosis process would be significantly slowed by high Reynolds number and turbulence (Re = 3,500 and 5,000). Specifically, the growth rate of the thrombus is found to be inversely related to the Reynolds number.

## Discussion

The thrombus formation has placed enormous strain on the human cardiovascular system and medical devices. Both blood properties and the flowing geometric environment have contributed to the thrombus issues. Prediction of the thrombosis process can play a key role in mitigating the risk of blood clots in humans and devices. The present study was designed to simulate the thrombus formation in the FDA benchmark nozzle by coupling the computational hemodynamics and thrombosis model.

Platelets are one of the important components of the thrombus and the thrombi would stop growing when the platelets are depleted. We propose a positive proportional relationship between the thrombus volume and platelets number based on the thrombosis experiment. In the present hemodynamics-based thrombosis model, platelet consumption is considered by using a simplified method.

Thrombus model validation is indispensable before application. Menichini and Xu (2016) compared the computational result of the thrombosis model with an early BSF experiment (Taylor et al., 2014). However, the blood used in the experiment comes from bovines. Data from the latest BSF experiment which used human blood shows the thrombi is a triangular ramp shape (Yang et al., 2020). While a quasi-triangular slope shape is observed in our simulation results, which is similar to the previous computational report (Yang et al., 2021). There may be three reasons for the difference in thrombus shape. First, it may be caused by the effect of the contrast agent injected into the blood flow during the experiment. Although the contrast agent does not affect the stability of the thrombus (Walvick et al., 2011), the edge of the thrombus has not yet reached a stable state and fragile edges may be destroyed. Second, the spatial resolution of the MRI is limited. The thrombus edges may be too thin to be captured by MRI technology. Finally, the simulated thrombus shape may be limited by the choice of platelet activation mechanism. We considered the contributions of resting platelets and thrombus exposure in the present study. However, the shear-induced platelet activation is also crucial (Holme et al., 1997). The hemodynamic-based thrombus model still needs to be improved.

In the present study, the SST model was applied to simulate the turbulent blood flow and the performance of this model was confirmed by the previous interlaboratory results (NCI Hub contributors, 2022). Additionally, our computational result is consistent with the previous report (Stewart et al., 2012). However, the blood flow details cannot be reproduced when the Reynolds number increased to 2000 and we skipped this special scenario. A recent reference (Manchester and Xu, 2020) reported the complicated transition process between laminar flow and turbulent flow in the FDA nozzle by using the LES method and the simulation results are satisfactory. The application of the LES model would be conducive to improving the agreement between the numerical simulation and the experimental measurement in this study.

We found that the turbulence has remarkable effects on the thrombus shape and simulation time, while the role of laminar flow is negligible. As the Reynolds number increases, the final thrombus shape changes from centrosymmetric to axisymmetric due to the presence of turbulence. Besides, the rate of thrombosis is significantly reduced and eventually stops when the Reynolds number is 6,500. This result may be explained by the fact that the low-SSR region to initiate coagulant disappeared. This phenomenon has two implications for us in avoiding thrombosis in medical devices. On the one hand, the low-wall shear stress region should be eliminated to reduce the thrombosis in the medical devices, and on the other hand, it should be noted that hemolysis would occur when the Reynolds number is high, and a balance between thrombosis and hemolysis can be sought by controlling turbulent flow.

This paper firstly reports the thrombus formation process in the FDA benchmark nozzle. However, the major limitation should be clarified. The thrombosis model was validated based on the BFS experiment, we adopted the same model and setup to predict the thrombus formation process in the FDA nozzle, while there is a lack of experimental measurement data to support our primary findings. Besides, the coagulation cascade involves a lot of biochemical species, such as thrombin, fibrin, and fibrinogen (Sarrami-Foroushani et al., 2019). Considering the complex chain of biochemical reactions would significantly slow down the simulated thrombosis process. Therefore, the *C* is applied to simplify the complex coagulation cascade in this hemodynamics-based thrombus model. However, the simulation strategy may reduce the accuracy to some extent. Finally, we adopted the SST model to simulate the turbulent blood flow. This model may neglect the complex fluctuating turbulent patterns, which have been reported in previous DNS/LES studies of the FDA nozzle (Fehn et al., 2019; Sánchez Abad et al., 2020). High-level turbulence models would be coupled with the thrombosis model to explore more details in our future study.

## Conclusion

This study firstly investigates the thrombosis process in the FDA benchmark nozzle by coupling the CFD method with the macro-scale thrombus model. We considered the effect of platelet consumption and the hemodynamic-based thrombus model was validated by referring to the latest experimental data. The annular thrombus was observed in the FDA nozzle. Besides, the accuracy of the SST model was confirmed in different turbulent flow conditions. We found that turbulence could change the shape of centrosymmetric thrombus to axisymmetric and high Reynolds number blood flow would delay or even prevent thrombosis, which confirms the opposing relationship between thrombosis and hemolysis. In conclusion, the thrombosis process in the FDA nozzle was predicted using the numerical simulation method and the effect of turbulence on the thrombosis was preliminary revealed.

## Data Availability

The original contributions presented in the study are included in the article/Supplementary Material, further inquiries can be directed to the corresponding author.
